# Comparison of inflationary non-invasive blood pressure (iNIBP) monitoring technology and conventional deflationary non-invasive blood pressure (dNIBP) measurement in detecting hypotension during cesarean section

**DOI:** 10.1186/s40981-017-0145-y

**Published:** 2018-01-05

**Authors:** Akiko Yamashita, Shingo Irikoma

**Affiliations:** 1 Division of Anesthesiology, Seirei Hamamatsu General Hospital, 1-12-12 Sumiyoshi, Hamamatsu City, 430–8558 Japan; 2 Division of Perinatology, Fetal Diagnosis and Therapy, Maternal and Perinatal Care Center, Seirei Hamamatsu General Hospital, 1-12-12 Sumiyoshi, Hamamatsu City, 430–8558 Japan

**Keywords:** Cesarean section, Maternal hypotension, Combined spinal-epidural anesthesia, iNIBP

## Abstract

**Background:**

The Nihon Kohden linear inflationary non-invasive blood pressure (iNIBP) monitoring technology is an oscillometric device that measures blood pressure by detecting oscillations during inflation. Systolic blood pressure can be recorded without overinflating the cuff higher than the true systolic pressure. Thus, total time taken for inflation and deflation is shorter than that by the conventional deflation devices. In this study, the ability of iNIBP to detect maternal hypotension during cesarean section faster than deflationary non-invasive blood pressure (dNIBP) monitoring devices under clinical settings was evaluated prospectively.

**Methods:**

A prospective study of singleton planned cesarean sections at a tertiary center was conducted from August 2015 to April 2016. The combined spinal-epidural anesthesia (CSEA) technique through a single puncture was performed for cesarean section at the center where the study was carried out. An iNIBP cuff was placed on the same arm as the intravenous line, and a dNIBP cuff was placed on the other arm. Due to left uterine displacement by approximately 10° tilt of OR table, hypotension in this study was defined as systolic pressure of 107 mmHg or less, when measured in the left arm, which was about 10 cm lower, and pressure of 92 mmHg or less in the right arm which was about 10 cm higher. This setup was done to evaluate which device detected hypotension faster under clinical settings. A two-tailed *Z* test was performed to statistically analyze the difference between iNIBP and dNIBP measurement results.

**Results:**

One hundred singleton planned cesarean deliveries under CSEA were included after 36 weeks of gestation. Out of the 100, 76 women (76%) experienced maternal hypotension. Of these, iNIBP detected hypotension faster than dNIBP in 47 cases (61.8%).

**Conclusion:**

It was found that iNIBP detected hypotension faster than conventional dNIBP without compromising the reliability of measurement. This may lead to early treatment of maternal hypotension and prevention of adverse events related to the mother and the fetus.

## Introduction

Nowadays, spinal anesthesia is the first choice for anesthesia during cesarean sections. Pregnant women tend to experience a drop in blood pressure because of supine hypotensive syndrome and decreased peripheral vascular resistance. Maternal hypotension is an adverse event for the mother and the fetus and can cause fetal bradycardia [1]. Therefore, it is important to detect maternal hypotension as soon as possible and control it promptly. However, it often takes time to measure blood pressure because of noise and change in body position in clinical practice.

The Nihon Kohden linear inflationary non-invasive blood pressure (iNIBP®) measurement technology is an oscillometric device used to measure blood pressure by detecting oscillations during inflation. We can read systolic blood pressures without the need to overinflate the cuff higher than the true systolic pressure. Thus, the total time taken for inflation and deflation is shorter than that taken by conventional deflationary devices.

In this study, we prospectively evaluated whether iNIBP can detect maternal hypotension during spinal anesthesia earlier than deflationary non-invasive blood pressure (dNIBP) in clinical settings in which noise and vibration could lead to inaccurate measurement, requiring re-measurement of blood pressure.

## Methods

Approval of the Institutional Review Broad and written informed consent from the patients were obtained for this study. We conducted a prospective study of singleton planned cesarean sections at a tertiary center from August 2015 to April 2016 (UMIN000027385).

At our center, the combined spinal-epidural anesthesia (CSEA) technique is performed for cesarean sections. The L3-4 interspace was selected. The CSEA was initiated with 0.5% hyperbaric bupivacaine (8 mg) and fentanyl (20 μg) via a 27-gauge spinal needle placed via a 16-gauge Tuohy needle, and the epidural catheter was inserted.

**Fig. 1:**
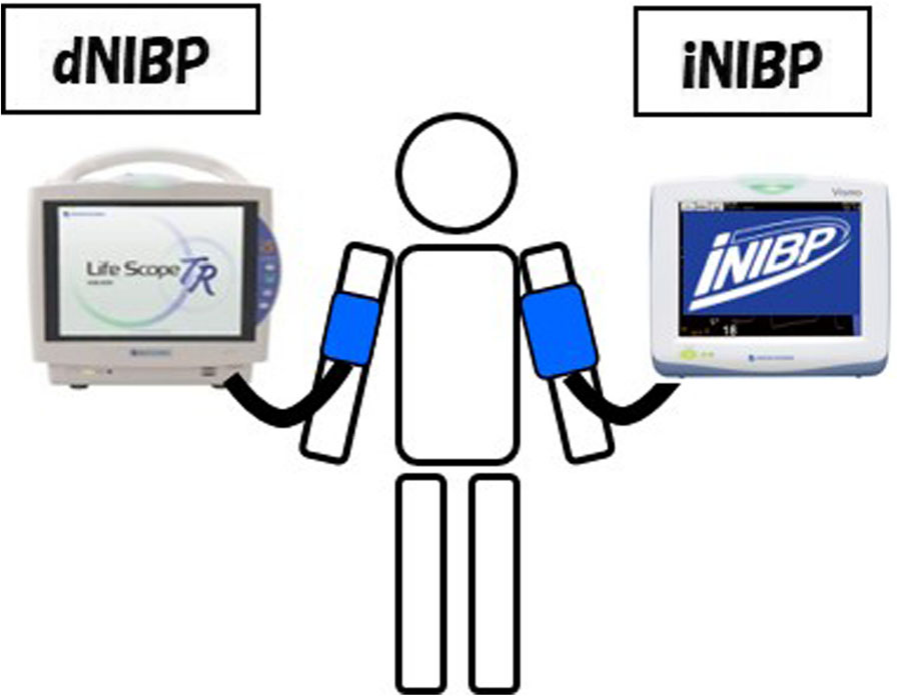
Blood pressure measurement. iNIBP cuff was wrapped around the same arm as the intravenous line and dNIBP cuff was wrapped around the other arm

**Fig. 2:**
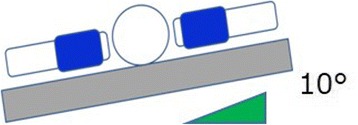
Image depicting left uterine displacement (about 10°) to prevent hypotension

The iNIBP® cuff was wrapped around the arm with an established intravenous line, and the dNIBP cuff was wrapped around the other arm (Fig. 1). Blood pressure was measured at 2-min intervals as per our routine, from beginning of spinal anesthesia to the delivery of the baby. To prevent hypotension, left displacement of the uterus (10°) was performed by tilting the OR table (Fig. 2). We defined hypotension as systolic arterial pressure of 107 mmHg or less on the left arm which is about 10 cm lower and pressure of 92 mmHg or less on the right arm which is about 10 cm higher. At the same time, we began to measure blood pressure by both devices and examined which could detect maternal hypotension faster.

A two-sided *Z* test was performed to statistically analyze the differences between the iNIBP and dNIBP measurements.

## Results

We included 100 singleton planned cesarean deliveries under spinal CSEA, after 36 weeks of gestation.

The demographic and clinical characteristics of the cases are described in Table 1. The average maternal age at pregnancy was 34.7 ± 5.0. The median gestational age at delivery was 37.9 weeks (range, 36–40). The average body mass index (BMI) was 24.7 ± 2.8. The reasons for undergoing cesarean section were found to be previous cesarean section in 72 cases (72%), non-vertex position in 15 cases (15%), placenta previa in 10 cases (10%), and other reasons in 3 cases.

**Table 1:** Maternal demographic and clinical characteristics

	Mean ± SD or median (range)
Age (years)	34.7 ± 5.0
GA at delivery (weeks)	37.9 (36–40)
BMI	24.7 ± 2.8

*GA* gestational age, *BMI* body mass index

Out of the 100, 76 women (76%) experienced maternal hypotension, as measured by iNIBP or dNIBP. Of these, iNIBP detected hypotension faster than dNIBP in 47 cases (61.8%) and dNIBP detected faster than iNIBP in 29 cases (38.2%). This comparison is shown in Table 2. It was found that iNIBP could detect maternal hypotension significantly earlier than dNIBP in clinical settings (*p* = 0.03).

**Table 2:** Comparison of the speeds of detection of hypotension by different devices

	No hypotension	iNIBP	dNIBP	*p* value
The number of cases in which hypotension was detected faster (*n* (%))	24	47 (61.8)	29 (38.2)	0.03

## Discussion

We conducted the prospective study to evaluate which method between iNIBP and dNIBP could detect the post-spinal maternal hypotension during cesarean section faster. It was found that iNIBP could detect maternal hypotension faster even under clinical settings, in which noise and vibration could lead to inaccurate measurement and require re-measurement of blood pressure.

The spinal anesthesia for cesarean section is likely to cause maternal hypotension because of the spine hypotensive syndrome and decreased peripheral vascular resistance. Hypotension during cesarean section is defined as systolic blood pressure ≤ 100 mmHg or ≤ 20% below the baseline blood pressure [2]. The sustained maternal hypotension affects not only maternal circulation but also uteroplacental blood flow. As it may cause fetal bradycardia and acidosis [1], it needs to be prevented and treated promptly. Left uterine displacement and fluid management by colloid co-loading are the preventive measures employed [3].

The conventional non-invasive blood pressure monitoring devices are designed to detect oscillations during deflation. Therefore, they require elevation of the cuff pressure higher than the true systolic pressure. This excessive cuff pressure may cause adverse events such as pain, venous stasis, thrombophlebitis, peripheral neuropathy, and compartment syndrome [4, 5]. Meanwhile, the iNIBP is designed to detect oscillations slowly during inflation and reduce the cuff pressure at the time of detecting the systolic blood pressure. Thus, it requires lower cuff pressure and shorter time to detect blood pressure as compared to dNIBP [6].

Furthermore, previous studies suggested that the hemodynamic changes caused by pregnancy and pre-eclampsia may attribute to inaccuracy of the devices. Immediate detection of the oscillations during inflation may provide accurate measurements without transmission of the pressure wave. Therefore, inflationary oscillometry may be more accurate than deflationary oscillometry and mercury sphygmomanometry in pregnancy and pre-eclampsia cases [7, 8].

A limitation of this study is its small sample size. Further research is required to have a clear understanding of the advantages of iNIBP for cesarean section cases. A further limitation was that blood pressure was measured not every minute but 2 min. The BP measurement every minute until delivery of the baby is recommended.

Through this study, we conclude that iNIBP detects hypotension faster than conventional dNIBP, without compromising the reliability of measurement. This may lead to early treatment of maternal hypotension and prevention of maternal and fetal adverse events.
